# Supplementary material for: A model of decentralized vision in the sea urchin Diadema africanum
Source: iScience. 2023 Feb 28;26(4):106295. doi: 10.1016/j.isci.2023.106295 (PMC10025101; doi:10.1016/j.isci.2023.106295)
Supplement: Document S1. Figures S1–S4, Tables S1–S3, and Data S1–S6 [file mmc1.pdf]

iScience, Volume *26*

Supplemental information

A model of decentralized vision in the sea urchin *Diadema africanum*

Tianshu Li, John Kirwan, Maria Ina Arnone, Dan-Eric Nilsson, and Giancarlo La Camera

A model of decentralized vision in the sea urchin *Diadema africanum*

Supplemental Information

Tianshu Li[a,b,c], John Kirwan[d,e], Maria Ina Arnone[d], Dan-Eric Nilsson[e], Giancarlo La Camera[a,b,c,*]

[a] Department of Neurobiology & Behavior, Stony Brook University, Stony Brook, NY, USA
[b] Program in Neuroscience, Stony Brook University, Stony Brook, NY, USA
[c] Center for Neural Circuit Dynamics, Stony Brook University, Stony Brook, NY, USA
[d] Stazione Zoologica Anton Dohrn, Naples, Italy
[e] Lund Vision Group, Department of Biology, Lund University, Lund, Sweden
[*] Lead Contact. Email: giancarlo.lacamera@stonybrook.edu

Data S1

Experimental setup of Kirwan *et al* (2018)

Here we first briefly describe the setup for the taxis experiments reported in Kirwan et al. (2018). Individual sea urchins of the species *D. africanum* were placed in a lit arena, surrounded by printed patterns containing a printed visual stimulus (see Figure 3 of the main text). The arena comprised a cylinder of transparent acrylic and was surrounded by a white cylinder to exclude external cues. An array of four equidistant clusters of LEDs resulting in broad-spectrum visible illumination were placed above the arena. A remote-controlled camera was attached in an opening in an illumination diffuser at the top of the arena and was used to record time-lapse videos at a rate of 5 frames per second. The arena was filled with filtered natural seawater, at the same temperature at which the animals were housed (20°C). In each trial, the animal was placed by hand in the center of the arena and allowed to move to the periphery. Each trial continued for a maximum of 6 min or until the animal approached the arena wall. A trial was deemed complete if the animal moved at least three-quarters of the radial distance between the center and arena walls. Trials were conducted in sets of four and the stimulus was moved 90° clockwise for each subsequent trial, to remove the influence of any non-visual directional cues. Sets with individuals for which there were fewer than four completed trials (e.g. due to a loss of motivation) were excluded from analysis. The base of the arena was cleaned between trial sets with a brush to obscure chemical cues and the water was partially or completely changed, depending on its clarity. Experiments were performed during the daylight period of their entrainment. The frame rate for the recordings was 1 frame/s. Full details can be found in Kirwan et al. (2018).

Data S2

Stimuli and coordinate systems

In the original experiments, the printed patterns surrounding the arena consisted of greyscale printed images, which were uniform in the vertical plane but in the horizontal plane included stimuli that consisted of dark regions set against a lighter background (Figure 3B of the main text). The main patterns used a bar stimulus and a difference of Gaussians (DoG). These stimuli were used in the experiments of Kirwan et al. (2018) analyzed here. The remaining stimuli were used in this paper to provide novel model predictions. In the bar stimulus, a region of homogeneously black stimulus was presented against a white background. In the DoG stimulus, the center of the stimulus was maximally dark, but of increasing reflectance towards the periphery of the stimulus (on the horizontal axis) and reaching the maximum achievable reflectance before darkening into the grey background. All stimuli were isoluminant with respect to the remainder of the patterns due to the lighter regions flanking the stimulus, i.e., it is not possible to detect the stimulus by simply comparing the radiance profile of different parts of the arena from the centre without having a spatial resolution equivalent to the arc subtended by the stimulus itself.

In the following we refer to the stimulus as the entire pattern surrounding the arena wall. The intensity of the stimuli along the longitudinal dimension (i.e., going from west to east on a horizontal plane) were indexed by an angle $\phi \in [0, 360)^\circ$, formed by an arbitrary reference line and the line connecting the center of the arena to the point of interest on the wall (we use degree (°) as the angle units in this paper). The normalized light intensity of a stimulus at ϕ, $X_0(\phi)$ (henceforth simply 'intensity'), indicates the reflectance of the stimulus at an angle ϕ from the center and varied linearly from 0.176 to 1 (Kirwan et

al., 2018):

$$X_0(\phi) = 0.824(1 - X_{ink}(\phi)) + 0.176, \tag{S1}$$

where X_{ink} was the ink value used in the actual experiments to print the images on the arena outer wall (Figure 3B of the main text: the ink value of black is 1, for white is 0). Since photoreceptor cells respond to light, we replaced X_{ink} with $1 - X_{ink}$ to convert the ink value to the intensity of light reflected by the stimulus in Eq. S1. Note that in Eq. S1 we have taken into account the relative reflectance of the paper (only a proportion of the light was reflected by the paper). To obtain X_{ink} values between 0 and 1, we started from the functions $X_{ink,raw}$ reported in Table S1, and then rescaled $X_{ink,raw}$ by a simple linear normalization:

$$X_{ink} = \frac{X_{ink,raw} - \min_\phi X_{ink,raw}(\phi)}{\max_\phi X_{ink,raw}(\phi) - \min_\phi X_{ink,raw}(\phi)}. \tag{S2}$$

In addition to the stimuli above, we also used a homogeneous stimulus of constant intensity $X_0(\phi) = 0.77$ as control (same as the intensity of the DoG stimulus away from its center).

VISUAL STIMULI

Bar	$X_{ink,raw}(\phi) = \begin{cases} 1 & \text{if } \phi \in [0, \frac{\phi_{stim}}{2}] \cup [360^\circ - \frac{\phi_{stim}}{2}, 360^\circ) \\ 0 & \text{otherwise} \end{cases}$
Difference of Gaussians (DoG)	$\sigma_1 = \frac{\phi_{stim}}{2\sqrt{2\ln 2}}$<br>$\sigma_2 = 2\sigma_1$<br>$X_{ink,raw}(\phi) = e^{-\frac{\phi^2}{2\sigma_1^2}} - \frac{\sigma_1}{\sigma_2} e^{-\frac{\phi^2}{2\sigma_2^2}}$
1st Hermitian wavelet	$D = \frac{2\phi_{stim}}{2\sqrt{3}\sqrt{2\ln 2}}$<br>$g(\phi) = e^{-\frac{\phi^2}{2D^2}}$<br>$X_{ink,raw}(\phi) = -g'(\phi)$
Flanked bar (FB)	$X_{ink,raw}(\phi) = \begin{cases} 1 & \text{if } \phi \in [0, \frac{\phi_{stim}}{2}] \cup [360^\circ - \frac{\phi_{stim}}{2}, 360^\circ) \\ 0 & \text{if } \phi \in [\frac{\phi_{stim}}{2}, \phi_{stim}] \cup \\ & [360^\circ - \phi_{stim}, 360^\circ - \frac{\phi_{stim}}{2}) \\ 0.5 & \text{otherwise} \end{cases}$
Haar wavelet	$X_{ink,raw}(\phi) = \begin{cases} 1 & \text{if } \phi \in [0, \frac{\phi_{stim}}{2}] \\ 0 & \text{if } \phi \in [360^\circ - \frac{\phi_{stim}}{2}, 360^\circ) \\ 0.5 & \text{otherwise} \end{cases}$
Morlet wavelet	$D = \frac{2\phi_{stim}}{2\sqrt{2\ln 2}}$<br>$X_{ink,raw}(\phi) = e^{-\frac{\phi^2}{2D^2}} cos(\frac{2\pi\phi}{\phi_{stim}})$

Table S1: **Mathematical definitions of the stimuli. Related to Figure 3B of the main text.** Definitions of ϕ_{stim} and $X_{ink,raw}(\phi)$ for all stimuli. See Supplemental Data S2 for details.

Note that each stimulus had an intuitively clear 'center' (Figure 3B), which was always aligned to the reference line (i.e., the center of the stimulus was always at $\phi = 0^\circ$ on the wall of the arena). The center of each stimulus was characterized by an arc width ϕ_{stim} which encloses the high amplitude region of the stimulus, surrounding the center. For example, in the case of the bar stimulus (see Figure 3B of the

main text), ϕ_{stim} corresponds to the arc width of the black region, while for the DoG it corresponded to the arc width of the distance between the two white maxima.

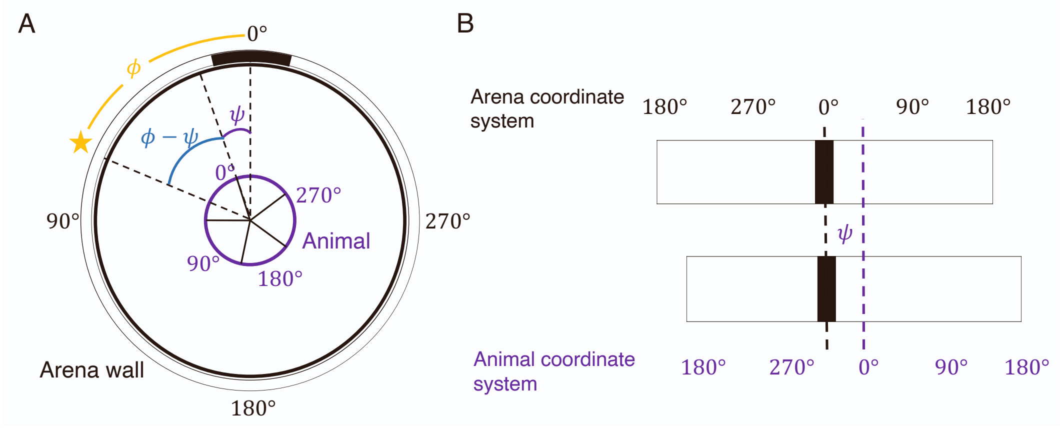

Figure S1: **Light intensity in the reference frame of the sea urchin. Related to Star Methods. A**. Diagram of a $\phi_{stim} = 40°$ bar stimulus attached to the arena outer wall (black circle). Black numbers outside of the arena indicate the arena's coordinate system. A sea urchin (purple circle) is placed at the center of the arena. Black solid lines originating from the center of the arena represent the ambulacra. One ambulacrum, here positioned at angle $\phi = 30°$, is randomly chosen to be the 'first ambulacrum' and is the reference direction in the animal's own coordinate system (purple numbers). The value of the ink at the location indicated by the yellow star is $X_{ink}(\phi) = 0$, and the light intensity at this location is $X(\phi - \psi) = X_0(\phi) = 0.824(1 - X_{ink}(\phi)) + 0.176 = 1$. **B**. The top band is the stimulus paper in A cut at 180° in the arena's coordinate system (black numbers at the top). The bottom band is the stimulus paper cut at 180° in the animal's coordinate system (purple numbers at the bottom).

We next define the intensity of the stimulus in the animals' own coordinate system, defined by the longitudinal angle ψ between the 'first ambulacrum' (arbitrarily chosen) and the origin of the arena's coordinate system. The angle ψ defines the orientation of the animal in the arena, which is assumed constant during motion (i.e., the animal does not rotate as it moves; see Supplemental Data S5 for details). Therefore, each animal has its own (constant) value of ψ. This is illustrated in Figure S1A, which shows a cartoon of the arena (black circle) with a sea urchin (purple circle) placed at the center of the arena. Black numbers outside the arena are coordinates in the arena's coordinate system, while purple numbers are the 'local' coordinates in the sea urchin's system. Given the local coordinate $\phi' = \phi - \psi$, the intensity of light at angle ϕ' is given by

$$X(\phi') = X(\phi - \psi) = X_0(\phi). \tag{S3}$$

In our model, this quantity was the input to the photoreceptor cells distributed on the tube feet of the animal (Eq. 4 of Star Methods).

Data S3

Analytical formula for the preferred directions of eONR cells

Here we show how to compute analytically the preferred directions of eONR cells required in the definition of the the population vector, Eq. 11 of Star Methods. To do so, we associate unique vectors to each PRC, RN neuron and eONR neuron, and in each case define their preferred direction as the orientation of the associated vector. We then show that this definition matches the definition of preferred directions of eONR cells given in the main text.

First, consider the unit vector $\hat{\boldsymbol{z}}_i^{k,PRC}$ associated to PRC i on ambulacrum k having direction of maximum sensitivity $\phi_{i,dms}^{k,PRC}$ (see Star Methods, Section "Photoreceptor cells"):

$$\hat{\boldsymbol{z}}_i^{k,PRC} = (\cos \phi_{i,dms}^{k,PRC}, \sin \phi_{i,dms}^{k,PRC}), \tag{S4}$$

$\hat{\boldsymbol{z}}_i^{k,PRC}$ is a vector with unit length and orientation $\phi_{i,dms}^{k,PRC}$.

We next associate a vector to each RN neuron by taking the linear combination of all PRC vectors connected to it, where the connection weights are used as coefficients of the linear combination:

$$\boldsymbol{z}_i^{k,RN} = \sum_j W_{RP}^k(i,j)\hat{\boldsymbol{z}}_j^{k,PRC} = \overline{W}_{RN}^k \sum_{j \in conn} \hat{\boldsymbol{z}}_j^{k,PRC}, \tag{S5}$$

where *conn* is the set of PRCs connected to the RN cell i on ambulacrum k, and we have also used the fact that the non-zero connections $W_{RP}(i,j)$ are all equal to the same value, here called $\overline{W}_{RN}^k$ (see Eq. 5 of Star Methods). Since the lateral connections among RN neuron groups are symmetric with respect to the mean direction of maximum sensitivity of afferent PRCs, the RN neurons inherit a preferred direction (the orientation of vector $\boldsymbol{z}_i^{k,RN}$) which is the same as the average direction of maximum sensitivity of its afferent PRCs.

We then associate a vector to each eONR cell in a similar way:

$$\boldsymbol{z}_i^{ONR} = \sum_{k,j} W_{OR}(i,j)\hat{\boldsymbol{z}}_j^{k,RN} = \overline{W}_{OR} \sum_{k,j \in conn'} \hat{\boldsymbol{z}}_j^{k,RN}, \tag{S6}$$

where $\hat{\boldsymbol{z}} \doteq \boldsymbol{z}/||\boldsymbol{z}||$, the sum over k is over the five ambulacra, and $conn'$ is the set of all RNs connected to the ith ONR cell via the 'indirect pathway' containing intermediate iONR cells (as shown in Figure 1B of the main text). Again, the lateral connections among groups of eONR neurons do not impact the direction of $\boldsymbol{z}_i^{ONR}$, and therefore they are not included in the definition of $\boldsymbol{z}_i^{ONR}$.

By definition, the preferred direction of eONR cells is given by the orientation of their associated vectors $\boldsymbol{z}_i^{ONR}$. Figure S2 shows that the preferred directions computed this way agree with the preferred directions obtained in response to a 2° white bar stimulus – the definition used in the main text.

Also note that $\phi_{i,pref}^{ONR}$ matches the orientation of the location of eONR cell i at the onset location of each ambulacrum $(0°, 72°, 144°, ...)$, and then a mismatch linearly accrues until the location of the next ambulacrum is reached. The mismatch is due to the lack of PRCs in the region between ambulacra, and its effect on the population vector is evident from the animations discussed in Supplemental Data S4.

We can now also write the population vector as a function of the PRC vectors. Recall that the population vector is defined as weighted sum of the eONR vectors (Eq. 11 of Star Methods), or, in the present notation,

$$\boldsymbol{v}_{pop} = \sum_i r_i^{ONR}\hat{\boldsymbol{z}}_i^{ONR}, \tag{S7}$$

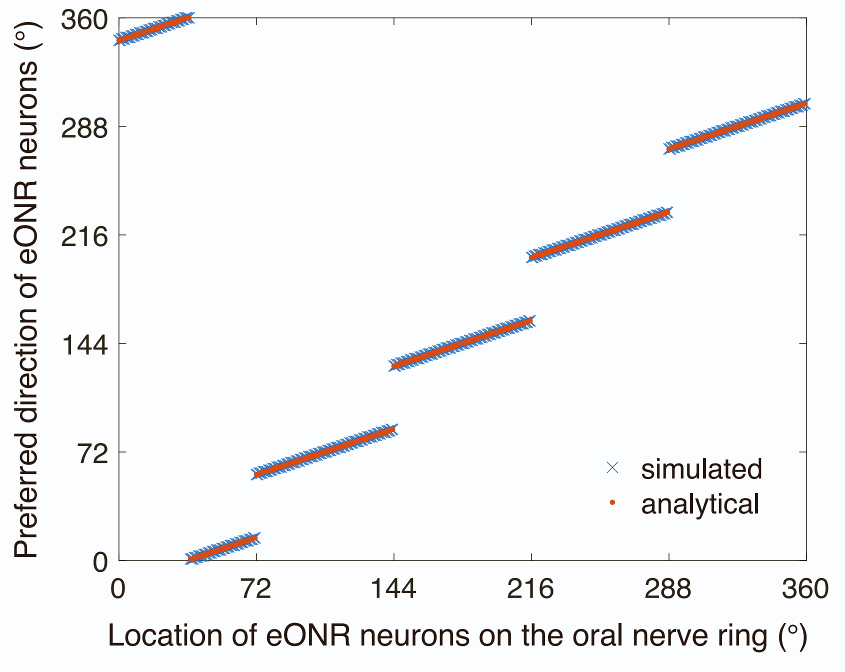

Figure S2: **Preferred direction of eONR cells. Related to Star Methods**. The figure shows the orientation of the vector defined by Eq. S6 (red circles) with the direction of a 2° white bar stimulus that causes the maximal increase in activity in the eONR cell in simulations of the model (blue crosses). The locations at $0°, 72°, 144°, 216°$ and $288°$ represent the locations of the ambulacra. Starting from each ambulacrum, the preferred direction is a linear function of the location of the eONR cell with slope smaller than 1. This figure confirms the correctness of the analytical formula, Eq. S6, for predicting the preferred direction of eONR cells (see the text for details).

where $\hat{\boldsymbol{z}}_i^{ONR}$ are the normalized vectors $\boldsymbol{z}_i^{ONR}/||\hat{\boldsymbol{z}}_i^{ONR}||$. Putting together the steps leading to Eq. S6, we obtain

$$\boldsymbol{v}_{pop} \propto \sum_{i=1}^{N_{ONR}} r_i^{ONR} \sum_{k,j \in \mathcal{C}(i)} \hat{\boldsymbol{z}}_j^{k,PRC}, \tag{S8}$$

a vector sum of PRC vectors $\hat{\boldsymbol{z}}_j^{k,PRC}$ weighted by coefficients proportional to the firing rates r_i^{ONR}. The sum over $k, j \in \mathcal{C}(i)$ is over all PRCs connected to eONR cell i via the 'vertical pathway' shown in Figure 1B of the main text including intermediate RN and eONR cells. We note that this formula holds for our model but it does not necessarily hold for a different (especially non-symmetrical) topology of the neural connections from PRCs to ONR.

Data S4

Neural activity and population vector as a function of the animal orientation

Table S2 lists the URL of three animations of the activity of PRCs, RN neurons, eONR neurons and the population vector as the relative position of the animal with respect to the stimulus changes. The stimuli used were the 40° bar, a 29° DoG and a 69° DoG. To better visualize this processs, we fixed the animal's position and rotated the stimulus, which is equivalent to fixing the position of the stimulus and rotating the animal. Note how the orientation of the animal with respect to the stimulus affects stimulus detection due to the lack of PRCs between ambulacra. For narrow stimuli, this results in the existence of effective 'blind spots' between ambulacra.

**Stimulus**	**URL**
29° DoG	https://www.dropbox.com/s/2cmcet380m1ph23/rotation_29DoG_rho30_delta15.mp4?dl=0
40° Bar	https://www.dropbox.com/s/m25m9pocdkgfhee/rotation_40bar_rho30_delta15.mp4?dl=0
69° DoG	https://www.dropbox.com/s/ujkhzhk3b5eln4i/rotation_69DoG_rho30_delta15.mp4?dl=0

Table S2: URLs of animations showing neural activity and population vector as a function of the relative orientation of the sea urchin and the center of the stimulus. Files also available from https://github.com/lacameralab/diadema/tree/main/diadema_animations.

Data S5

Simulation of behavioral trajectories

It is convenient to describe the model in the reference frame of the moving animal, where the location on the animal is described by an angle ϕ from its reference 'first' ambulacrum (see Supplemental Data S2). Each behavioral trial started with the animal located at the center of the arena with a random orientation ψ with respect the center of the stimulus (see Figure S1). Given a stimulus $X_0(\hat{\phi})$ at location $\hat{\phi}$ on the arena wall, the input to the PRCs was $X(\hat{\phi} - \psi) \equiv X(\phi)$ in the coordinate system of the sea urchin (this is Eq. S3 after a change of notation $\phi \to \hat{\phi}$ and $\phi' \to \phi$). We scaled the radius of the arena to be 1 and discretized the movement of the animal to occur in small steps $\delta r = 0.1$ at discrete points in time. Thus, rather than moving straight to the wall, the animal only made a small step and established the direction of the next step according to the stimulus detected in the current position. The length of each step roughly mimics the number of steps required to reach the wall of the arena during the experiments. We also assumed the animal does not rotate during movement as observed in experiment, in other words, the orientation of the unit vectors $\hat{\boldsymbol{e}}_1$, $\hat{\boldsymbol{e}}_2$ specifying the coordinate system of the animal (Figure S3) does not change during locomotion, which empirically seems, at least approximately, correct. After each step, we updated the position of the animal by sampling the new position from the mixture of Gaussian distributions, Eq. 13 of Star Methods.

The procedure is illustrated in Figure S3. Starting at the center of the arena and facing stimulus X, the animal moves to a new position $\boldsymbol{\delta r}$ along, say, direction α: $\boldsymbol{\delta r} = (\delta r \cos\alpha, \delta r \sin\alpha)$. The intensity of the stimulus at the representative location $\boldsymbol{x}$ of the yellow star (in the coordinate system of the animal), is now detected at position $\boldsymbol{x} - \boldsymbol{\delta r}$, forming an angle ϕ_{next} from the first ambulacrum. Given $\boldsymbol{x} = (\cos\phi, \sin\phi)$

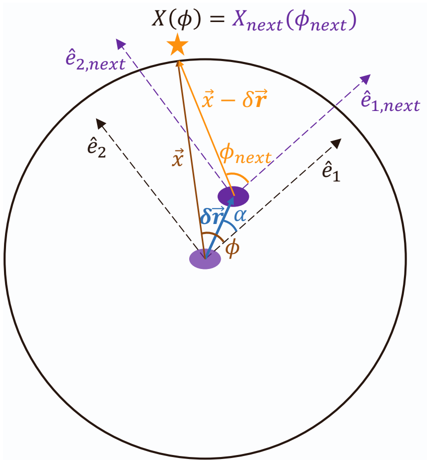

Figure S3: **Stimulus coordinates during simulation of movement. Related to Star Methods.** The unit vectors $\hat{\boldsymbol{e}}_1$ and $\hat{\boldsymbol{e}}_2$ are the principal axes of the coordinate system of the sea urchin (purple ellipsoid). $\hat{\boldsymbol{e}}_i$ is the direction of the first ambulacrum in the coordinate system of the arena. The animal moves without rotating, so that the orientation of its axes remains constant (i.e., $\hat{\boldsymbol{e}}_{1,next} = \hat{\boldsymbol{e}}_1$ and $\hat{\boldsymbol{e}}_{2,next} = \hat{\boldsymbol{e}}_2$). The relative position of the yellow star with respect to the sea urchin is at ϕ when the animal is at the center of the arena, and at position ϕ_{next} after the animal has made one step $\boldsymbol{\delta r}$. From the new position, the stimulus appears as a new stimulus X_{next}, which is then used to compute the next movement, and so on until the animal reaches the wall of the arena and its final position is recorded.

and $\boldsymbol{x} - \boldsymbol{\delta r} = (\cos\phi - \delta r \cos\alpha, \sin\phi - \delta r \sin\alpha) \doteq (v_x, v_y)$, the new angle ϕ_{next} is given by

$$\phi_{next} = \tan^{-1}\left(\frac{v_y}{v_x}\right) \quad (+180^\circ \text{ if } v_x < 0), \tag{S9}$$

and the yellow star is detected as having intensity $X_{next}(\phi_{next})$ from this new position. Note that in this new position, a different point on the wall faces the animal in the direction parallel to the $\boldsymbol{x}$ vector, that is, the stimulus appears as a new stimulus X_{next}, which is then used to compute the next movement. The trial ended when the animal reached the wall of the arena. Since *D. africanum* has long spines, we modeled its radius as being 1/4 of the distance between the wall and the center of the arena. When the center of the sea urchin reached a distance 3/4 from the center of the arena, the animal had reached the wall and the simulation stopped. The current position at this point was projected orthogonally on the arena wall and logged as the final position.

**Stimulus**	**URL**
29° DoG	`https://www.dropbox.com/s/x9jbp04g0hpkjx7/trajectory_29DoG_N10_rho30_delta15.mp4?dl=0`
40° Bar	`https://www.dropbox.com/s/eh1jlk3n9fpzkez/trajectory_40bar_N10_rho30_delta15.mp4?dl=0`
69° DoG	`https://www.dropbox.com/s/fylhsr1a7oeaayf/trajectory_69DoG_N10_rho30_delta15.mp4?dl=0`

Table S3: URLs of animations showing simulations of the behavior of the model in the presence of a 40° bar, a 29° DoG and a 69° DoG. All tracks comprising 100 simulations per stimulus are shown in Figure 5 of the main text. Files also available from `https://github.com/lacameralab/diadema/tree/main/diadema_animations`.

Table S3 includes the URLs of three animations of the behavior of the model in response to a 40° bar, a 29° DoG and a 69° DoG. The urchin is represented by the moving circle with the 5 ambulacra drawn inside. The wall of the arena is represented by the large circle. The circle with the stimulus drawn on it and moving together with the urchin illustrates how the stimulus is 'seen' by the sea urchin at each new position. The red circle marks the threshold for the population vector (the latter always drawn in blue form the center of the arena). Each simulated trial ends when the edge of the urchin's body (the smaller circle) makes contact with the arena.

Data S6

The effect of variability in PRCs' acceptance angles

The results of the main text (summarized in Figure 6) were obtained using a constant acceptance angle for PRCs. Here we repeat the analysis of Section "Effect of location and acceptance angle of PRCs on spatial vision" of the main text and show that our results also hold in the case of random distributions of acceptance angles across PRCs. For concreteness, we chose, in each case, Gaussian distributions with mean $\langle \Delta\rho \rangle$ and standard deviation equal to $\langle \Delta\rho \rangle/4$ (different choices did not qualitatively alter the results). The results are shown in Figure S4. Except for noisier contours, the regions of parameter space defined by the $v_{max} \geq 4$ or $v_{max} \geq 5$ appear unchanged compared to those shown in Figure 6 of the main text for constant $\Delta\rho$.

References

Kirwan JD, Bok MJ, Smolka J, Foster JJ, Hernández JC, Nilsson DE (2018) The sea urchin diadema africanum uses low resolution vision to find shelter and deter enemies. *J Exp Biol* 221.

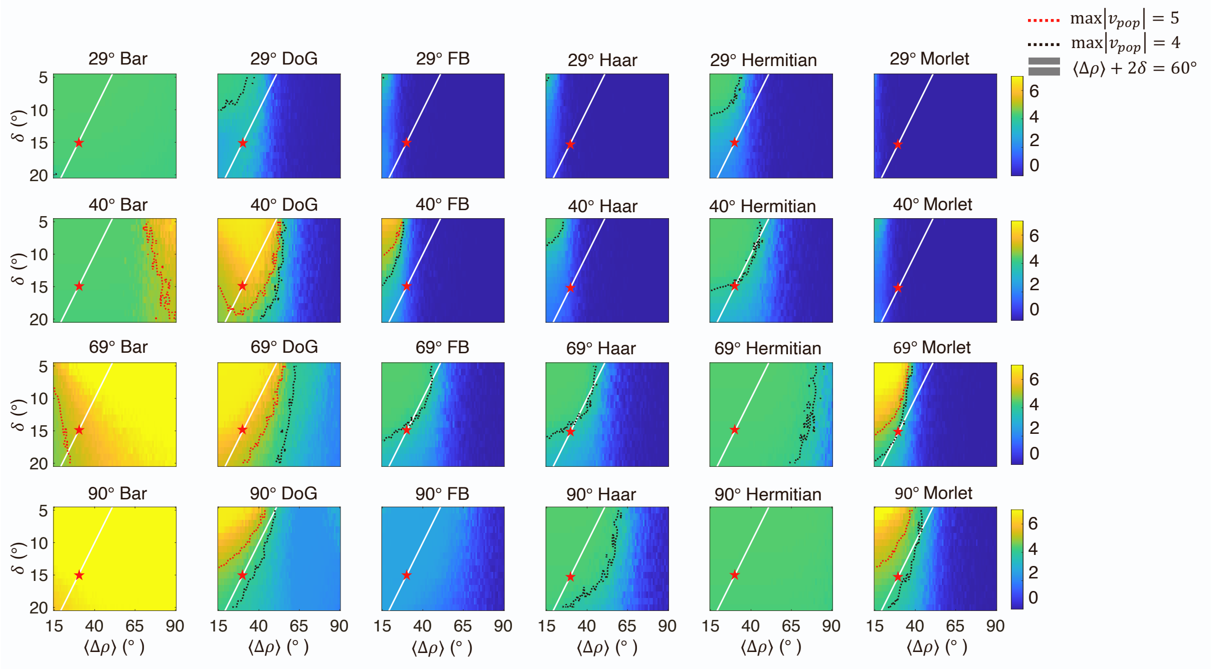

Figure S4: **Effect of acceptance angle and location of PRCs on the model's spatial vision. Related to Figure 6 of the main text.** Same as Figure 6 of the main text but with a random distribution of PRC acceptance angles $\Delta\rho$, chosen to be a Gaussian distribution with mean $\langle\Delta\rho\rangle$ and standard deviation $\langle\Delta\rho\rangle/4$. Each panel shows a heat map of v_{max}, the maximal length of the population vector across initial orientations of the animal, for a given stimulus and a given pair of values for $\langle\Delta\rho\rangle$ and δ. Each column shows the same stimulus for different arc widths of the target region (i.e., for different ϕ_{stim}, see Table S1), while each row shows the same ϕ_{stim} across different stimuli. If $v_{max} < \theta_p$, the animal cannot detect the stimulus from any orientation. The red dotted line is the contour line where $v_{max} = \theta_p$, while the black dotted line is the contour line where $v_{max} = 4$. The white line is the collection of points with $\langle\Delta\rho\rangle + 2\delta = 60°$. Red stars mark the point $(\langle\Delta\rho\rangle, \delta) = (30°, 15°)$, the parameter values used in the main simulations (where, however, $\Delta\rho$ was constant across PRCs). *DoG*: Different of Gaussians; *FB*: Flanked bar.
